# The impact of COVID-19 (Coronavirus) on children and young people with Down syndrome in the United Kingdom

**DOI:** 10.3389/fpsyg.2023.1175636

**Published:** 2023-06-02

**Authors:** Emma Pagnamenta, Penny Hodgkinson, Rosemary Davidson, Victoria L. Joffe

**Affiliations:** ^1^School of Psychology and Clinical Language Sciences, University of Reading, Reading, United Kingdom; ^2^School of Health and Social Care, University of Essex, Colchester, United Kingdom; ^3^Institute for Health Research, University of Bedfordshire, University Square, Luton, United Kingdom

**Keywords:** Down syndrome, COVID-19, mental health, communication, education, healthcare, speech and language therapy

## Abstract

The COVID-19 pandemic had a profound impact across the globe. Evidence suggests children with Special Educational Needs and Disabilities and their families experienced impacts on well-being and disruptions in support from education and health services. This study investigated the impact of measures associated with the COVID-19 pandemic on children and young people (CYP) with Down syndrome in the United Kingdom, specifically changes in speech, language and communication abilities, behavior, social, emotional and mental health and access to education and healthcare services. Forty-six parents/carers of CYP with Down Syndrome (aged 2–25 years) completed an online survey between June and September 2020. Parents/carers frequently reported deterioration in speech, language and communication, literacy and attention skills since the onset of the pandemic. Deterioration in social and emotional wellbeing and behavior, including greater reliance on adults were also reported for some CYP with Down syndrome. Parents reported challenges with home-schooling and reductions in support from education and community services. Preferences for support during COVID-19 were for professional support or from other parents. These findings have implications for the support that is now needed for CYP with Down syndrome and their families and for periods of social restrictions in the future.

## 1. Introduction

The COVID-19 pandemic has had a profound impact across the globe, including increased levels of anxiety and stress in the population (Salari et al., 2020; Shevlin et al., 2020) and consequences for the social and emotional wellbeing of children and young people (CYP) (Ma et al., 2021; Racine et al., 2021). In the United Kingdom, measures were introduced from March 2020 resulting in school closures and national and regional lockdowns, reducing time spent outside of home and interactions with others outside of immediate families. These measures continued until December 2021 (Institute for Government Analysis, 2022).

Evidence suggests that CYP with Special Educational Needs and Disabilities (SEND, an accepted term used in education in the United Kingdom) and their families may have been disproportionately affected by COVID-19 measures. In data collected early in the first United Kingdom lockdown (March–April 2020) parents of children with SEND reported raised levels of anxiety, stress and low mood in both themselves and their children, in addition to fears and worries specifically related to their child’s SEND (Asbury et al., 2021). Data collected at a similar time provides further evidence to suggest parents/carers of CYP with SEND were directly affected by COVID-19 measures. A survey of 415 parents/carers carried out in April 2020 in a region of England reported by O’Hagan and Kingdom (2020) found that 65% were physically and mentally exhausted, and 51% reported increased anxiety and depression. Subsequently, Sideropoulos et al. (2022a) compared parental report of anxiety and worries for their child with SEND (*n* = 407) and their neurotypical siblings to examine how COVID-19 measures affected young people with SEND differently. Data in relation to individuals across a wide age-range (1–45 years) including a range of diagnoses and a high prevalence of intellectual disabilities (76%), indicated raised anxiety in children with SEND in the first few months of the pandemic, as compared with their siblings. Awareness of COVID-19 and parents with greater anxiety predicted higher reported anxiety levels for individuals with SEND.

Families have also been impacted by changes and/or interruptions in their access to education and health services. Children and young people with SEND typically access support from multiple services, many of which were disrupted by the pandemic (Jeste et al., 2020). Major changes in the delivery of community children and young people’s services were reported, in part due to re-deployment of staff to support an urgent pandemic response (Chadd et al., 2021; NHS Confederation and NHS Providers, 2022). For example, many children received reduced speech and language therapy during lockdown, putting them at increased risk of poor academic attainment, and difficulties with friendships, social functions and mental health (Royal College of Speech and Language Therapists, 2021). Collating the data from three United Kingdom surveys of almost 5,500 parents/carers with children with SEND, O’Hagan and Kingdom (2020) reported the following themes: (1) reduction in external support from schools and therapy services, (2) parents and neurotypical siblings providing more care to their child/sibling with SEND, and (3) difficulties managing home learning due to school closures. Toseeb et al. (2020) conducted a survey of 339 parents/carers of CYP (aged 5–18 years) with SEND between March and May 2020 and found that less than half of parents of children with SEND reported that the support they received during the first lockdown was sufficient to meet their child’s needs. Interestingly however, there is also some evidence that school closures and lockdowns may have been associated with positive impacts for some children with SEND. In a survey carried out in June 2020 with ~1000 parents/carers, 38% reported an improvement in their child’s anxiety during the first lockdown, with reasons for this including less pressure, a less formal learning environment at home, better understanding of the child’s needs and fewer sensory issues (Special Needs Jungle, 2020).

Down syndrome is a common cause of SEND, with a prevalence of 25.4 per 10,000 total births in England (National Congenital Anomaly and Rare Disease Registration Service, 2019) and a population prevalence estimated at 10,438 children aged 0–18 years in England and Wales (Wu and Morris, 2013). Down syndrome is associated with a specific cognitive profile, including intellectual disability, and difficulties related to speech, language and communication, attention and executive function, with the level of need increasing as children reach school-age (Grieco et al., 2015). Relative strengths have been observed in receptive language and social use of communication (relative to mental age) with particular needs in the domains of expressive language (grammar and syntax) and phonology (Abbeduto et al., 2007; Laws et al., 2015). Down syndrome is also associated with a specific profile of literacy skills, and while strengths in reading and in particular word identification skills have been reported, there is also evidence that CYP with Down syndrome have difficulties with phonological awareness and decoding (Snowling et al., 2008; Loveall and Barton-Hulsey, 2021). There is however variability in the strengths and needs of children with Down syndrome. A recent exploratory study of 72 children reported multiple cognitive profiles: (1) similar levels of verbal and nonverbal abilities, (2) specific needs in the verbal domain relative to non-verbal abilities, and conversely: (3) more profound needs in nonverbal abilities as compared with verbal abilities (Onnivello et al., 2022). Children with Down syndrome are also reported to have a specific profile related to social, emotional and behavioral functioning. There is some evidence from a study of 8-year-olds that children with Down syndrome have lower levels of anxiety, when compared with data from 8-year-olds in the general population (Van Gameren-Oosterom et al., 2011). Strengths have been reported in pro-social behaviors alongside a higher risk of externalizing behaviors at school-age (e.g., impulsivity, disruptive behaviors) and higher rates of internalizing difficulties emerging later such as anxiety, depression and withdrawal, relative to other children with intellectual disability (Grieco et al., 2015).

There has been limited research focused on the impact of COVID-19 on CYP with Down syndrome specifically. Studies of CYP with SEND have included a broad range of different diagnoses, and while parents/carers of CYP with Down syndrome were included in some studies, these amounted to less than 3% of the total sample (less than 10 parents/carers; Toseeb et al., 2020; Asbury et al., 2021). A larger number of parents/carers of children with Down syndrome were recruited by Sideropoulos et al. (2022a) (*n*=103, 26% of the total sample) but no syndrome-specific conclusions can be drawn from these findings due to the heterogenous nature of the sample in terms of types of SEND. Data from a sample of adults with Down syndrome in Italy taken before the pandemic and during lockdown measures suggests a range of impacts, including a decrease in independence, increase in social withdrawal, decrease in aggressive behavior, and an increase in depressive symptoms (Villani et al., 2020). To our knowledge, only one study has reported on the impact of COVID-19 on CYP with Down syndrome. Sideropoulos et al. (2022b) compared data from 115 caregivers of CYP with Down syndrome with data from caregivers of children with different types of SEND before the pandemic, during the first lockdown (March 2020) and in January–March 2021 in the United Kingdom. Reported anxiety levels were higher during the pandemic than pre-pandemic levels for both groups, but lower overall for participants with Down syndrome. Awareness of COVID-19, health problems and diagnoses of anxiety disorder were predictors of anxiety for participants with Down syndrome and with other types of SEND.

This study aimed to extend findings reported previously by investigating the impact of measures associated with the COVID-19 pandemic on CYP with Down syndrome in the United Kingdom, specifically investigating changes in speech, language and communication abilities, behavior, social and emotional functioning, and mental health and access to education and healthcare services.

## 2. Materials and methods

### 2.1. Participants

Parents/caregivers of 151 children and young people (CYP) aged 2 to 25 years with speech, language and communication needs (59% male, mean age 9.9 years ranging from 2 to 25 years) from across the United Kingdom were recruited through parent networks and support groups (e.g., Mumsnet, Facebook groups, university parent and family networks), speech and language therapy networks and social media as part of a larger study investigating the impact of COVID-19 on CYP with speech, language and communication needs. No incentives were offered for participation. For the purposes of this study, participants were included if their child had a diagnosis of Down syndrome (*n* = 50). Four respondents were from the United States and were excluded.

### 2.2. Materials and procedure

Parents/caregivers completed an anonymous online survey via Qualtrics between 26^th^ June and 31^st^ September 2020. This followed the first United Kingdom lockdown, which was from 23rd March 2020 to the end of June 2020. The survey included a combination of multiple choice, Likert scale and open-ended questions in 5 sections: (1) demographic information about the parent/caregiver and the child (12 questions), (2) information about the child’s SEND, speech, language and communication needs and specialist support in place (4 questions), (3) support from education and healthcare services since the onset of the COVID-19 pandemic (5 questions), (4) impact of COVID-19 on children’s speech, language and communication abilities, social, emotional and behavioral functioning (10 questions), and (5) impact of COVID-19 on access to education and healthcare services and support needs (5 questions). Two further questions were included to check attention and appropriate responses to mitigate for fraudulent responses (Q13 and Q23, see Supplementary materials). The survey took approximately 20–30 min to complete, and parents had a week to complete the survey after starting (see Supplementary materials for the full survey).

The survey was piloted with five parents/caregivers of children with SEND and five professionals working with children and young people with SEND. Overall, participants reported they understood the questions and felt the length of the survey was appropriate but remarked that the sequencing of questions did not always flow logically. In response to this feedback, the questions were re-ordered and grouped into themes. Clarification was also requested for a few questions, and these were then rephrased to make them clearer on first reading.

### 2.3. Ethics statement

Ethical approval was granted by the Faculty of Science and Health Ethics Sub-Committee at the University of Essex before the start of the study. Respondents completed online consent prior to completing the survey and were reminded that their participation was voluntary and they were free to withdraw from the study at any time. Consent was required in order to proceed with the questionnaire.

### 2.4. Data analysis

#### 2.4.1. Analysis plan

The data collected was predominantly quantitative. Qualtrics, the online survey platform was used to host the questionnaire. Inbuilt into the platform is bot detection software which was enabled to identify multiple responses, and the settings were also changed to prevent multiple submissions from one respondent in addition to monitoring respondent metadata to determine if the same respondent was completing the survey on multiple occasions. The survey responses were then inputted to IBM SPSS Statistics for Windows, Version 27.0 (IBM Corp, 2020). A missing value analysis identified any additional inconsistencies and ensured that data categories were re-labelled where appropriate (i.e., changes from ordinal to nominal).

As the study was investigating the impact of COVID-19 on (a) speech, language, communication, behavior, social and emotional functioning, and (b) access to education and healthcare services for United Kingdom-based children and young people with Down syndrome the survey questions were designed to explore each of these aspects in detail. The analysis plan then centered around addressing these different domains by descriptively analyzing the proportions of respondent answers. The survey included free text boxes where, (a) respondents could write additional information if the options listed did not cover their experience, or (b) to give additional detail to a previous question. For example, ‘Was it more difficult to book health/well-being appointments since the onset of COVID-19?’, a yes/no answer, was followed up with a question asking *why* it was more difficult if respondents had answered ‘yes’. This served to give more detail and nuance to the set survey answers, but were not in-depth responses as characterized by qualitative research. Consequently, qualitative methods such as narrative analysis, discourse analysis or grounded theory were excluded in favor of a content analysis approach more suited to short form answers. The qualitative content analysis then supplemented the quantitative data, which answered the principal research questions. The content analysis plan was approached thus: Systematic reading of all free text responses per survey question; Formulation and quantification of nature of responses; Weighting of responses by frequency of use; Construction of themes and cross-checking with the research team; Discussion of findings, noting both commonalities in response and outlying experiences.

#### 2.4.2. Data analysis

Quantitative responses were analyzed using descriptive statistics including frequency information. Open-ended responses were analyzed using content analysis. Content analysis was used as a way to ‘organize and elicit meaning’ from the free text responses (Bengtsson, 2016), and to provide more detail and depth to the predominantly quantitative survey data collected as part of this study. A manifest analysis approach was employed, where researchers describe what respondents say and quote verbatim, as opposed to the more interpretative latent analysis, where the underlying meaning of a text is sought (Bengtsson, 2016). The authors were all involved in the content analysis process; as different stages were completed, results were discussed in context of the survey findings. Key themes arising from the analysis are reported, along with less dominant, but nevertheless notable insights.

## 3. Results

### 3.1. Demographic information

Forty-three participants (93%) were mothers (1 father, 1 grandparent, 1 other) and 43 (93%) were of white ethnic background (2 mixed race, 1 Chinese/Chinese British). Twenty-four (52%) were employed. Total household income ranged from less than £16,000 to more than £120,000. Ten (22%) participants were from households with a total income below the poverty threshold for 2020–21 (Joseph Rowntree Foundation, 2023).

The mean age of the CYP with Down syndrome was 10.2 years (range 2–25 years: nine aged 2–5 years; eighteen aged 6–11 years; twelve aged 12–18 years; three aged 19–25, and; four undisclosed). Twenty-three (50%) of the CYP with Down syndrome were male. Forty-two (91%) of the CYP were of white ethnic background (3 mixed race, 1 other) and 37 (80%) had siblings. Families responded from all regions of England (*n* = 32, 70%), Northern Ireland (*n* = 8, 17%), Ireland (*n* = 3, 6.5%) and Wales (*n* = 3, 6.5%). Five families (11%) reported speaking a language other than English at home (3 Spanish, 1 Afrikaans, 1 Portuguese).

Twenty-seven (59%) of the children were reported to have a diagnosis of Down syndrome only, with the remaining 19 (41%) having multiple SEND diagnoses (see Table 1). Twelve parents reported their child had a diagnosis of learning disabilities (26%) and 17 reported some form of sensory impairment (37%). There was a high prevalence of pre-existing speech, language and communication needs reported (present prior to the pandemic). The most common were difficulties with speech sounds (*n* = 42, 91%), expressive language difficulties (*n* = 34, 74%), reading and writing (*n* = 29, 63%), receptive language difficulties (*n* = 18, 39%), and stammering (*n* = 10, 22%). Pre-existing social, emotional and behavioral difficulties were also reported by some parents (see Table 1).

**Table 1:** Co-occurring pre-existing special educational needs and disability diagnoses and difficulties reported by parents.

SEND diagnosis	Frequency	Percent
Hearing impairment	9	19.6
Visual impairment	6	13.0
Autistic spectrum disorder	6	13.0
Developmental language disorder	5	10.9
Moderate learning disability (MLD)	5	10.9
Language disorder	4	8.7
Profound and multiple learning difficulty (PMLD)	4	8.7
Dyspraxia	3	6.5
Severe learning disability (SLD)	3	6.5
Attention deficit hyperactivity disorder	2	4.3
Multi-sensory impairment	2	4.3
Social communication disorder	2	4.3
Other	1	2.2
Speech, language and communication difficulty		
Difficulties with pronunciation/production of speech sounds	42	91.3
Difficulties with talking–using words and sentences	34	73.9
Reading and writing	29	63.0
Difficulties with understanding what other people say	18	39.1
Stammering/stuttering/dysfluency	10	21.7
Voice–affecting the voice box and how the voice sounds	3	6.5
Social, emotional and behavioral difficulty		
Social interaction difficulties	14	30.4
Behavior problems	10	21.7
Emotional difficulties	7	15.2

CYP with Down syndrome had a range of educational placements, including nursery (*n* = 5, 11%), mainstream school (*n* = 20, 43%) and specialist school (*n* = 15, 33%) settings (see Table 2). Forty (87%) had an Education, Health and Care Plan (a legal document in the United Kingdom describing a child’s SEND and the support needs of the child).

**Table 2:** Educational setting (please note two participants reported more than one educational setting).

Educational setting	Frequency	Percent	Attending school during pandemic	% Attending school
Nursery	5	10.9	1	20.0
Mainstream primary	17	37.0	4	23.5
Mainstream secondary	3	6.5	1	33.3
Special school	14	30.4	5	35.7
Further education college	3	6.5	0	0.0
Specialist independent college	1	2.2	0	0.0
Adult services/community	2	4.3	0	0.0
Home educated	1	2.2	0	0.0
No school place	2	4.3	0	0.0

### 3.2. Awareness of COVID-19

Overall, a relatively low level of awareness of COVID-19 was reported: 33 of the children (79%) were reported to rarely or never ask questions about COVID-19 and only 15 parents/caregivers (36%) agreed that their child was very aware of COVID-19. Parents reported a low frequency of child worries specifically about getting COVID-19 (*n* = 4, 9%) or leaving the house (*n* = 2, 4%). Few parents reported that their child was experiencing fears in this period (losing friendships *n* = 5 11%, getting ill *n* = 3 7%, losing parents *n* = 3 7%, losing grandparents *n* = 2 4%, dying n = 1 2%). Most parents/caregivers reported that it was difficult to explain COVID-19 to their child (*n* = 41, 89.2%), and a variety of resources were drawn upon to do so - most commonly social stories (*n* = 15, 32.6%), telling stories (*n* = 11, 23.9%), information from teachers/schools (*n* = 13, 28.3%) and their children talking with friends (*n* = 9, 19.6%).

### 3.3. Impacts of COVID-19 measures on CYP with Down syndrome

Twenty-eight respondents (67%) reported concerns that their child’s communication, learning and development had deteriorated since the onset of the pandemic. Table 3 shows that deterioration in communication, social/emotional skills, attention and learning, play, skills for daily living, and physical health were reported with the most frequently cited areas of deterioration being social skills (*n* = 20, 43.5%), speech sounds (*n* = 15, 32.6%), communication (*n* = 14, 30.4%), attention (*n* = 13, 28.3%), and using words and sentences (*n* = 12, 26.1%). Deterioration in physical skills, voice, play and stammering were rarely reported across the sample. A high level of parental concerns relating to the CYP with Down syndrome were reported, most commonly in social interactions (*n* = 40, 87%), meeting learning/development needs (*n* = 38, 82.6%) child health (*n* = 36, 78.2%), speech, language and communication development (*n* = 36, 78.2%) and emotional wellbeing (*n* = 36, 78.2%).

**Table 3:** Areas of deterioration in children as noticed by parents during the COVID-19 pandemic.

Area of deterioration	Frequency	Percent
**Communication**		
Communication	14	30.4
Speech/pronunciation/production of speech sounds	15	32.6
Stammering/stuttering/dysfluency	4	8.7
Language/understanding what other people say	6	13.0
Language/talking–using words and sentences	12	26.1
Voice	1	2.2
**Social/emotional**		
Social skills	20	43.5
Behavior	5	10.9
Emotional wellbeing	10	21.7
**Attention and learning**		
Attention	13	28.3
Memory	5	10.9
Reading and writing	10	21.7
**Play**	4	8.7
**Skills for daily living**	6	13
**Physical health**		
Physical difficulties impairing their ability to walk and/or move and/or talk	1	2.2
Physical health	1	2.2
**Other**		
Numeracy	1	2.2

Ten (24%) parents/carers reported an increase in anxiety in CYP with Down syndrome. Parents/carers conveyed a range of increased or new adverse behaviors in their child since the onset of the pandemic, most frequently increased reliance on television (*n* = 22, 48%), reduced motivation (*n* = 21, 46%), loneliness (*n* = 18, 39%), and greater dependence on adults (*n* = 17, 37%). Parents expanded on their responses in the free text boxes, describing the effects of isolation and anxiety as their children missed their routines, school friends and extended families. A parent of an only child described how her daughter had not seen other children for months curtailing any meet ups with school friends:

“she doesn’t understand social distancing and will hug strangers in public places.” (R9: 93)

Others recounted similar experiences, with children missing friends and social activities such as going to the cinema and discos. The lack of social interaction meant that social skills had been forgotten:

“Speech and language is my child’s main challenge but she needs face-to-face support which hasn’t been possible […] her need to socialise with other children has been a daily struggle.” (R174: 91)

“Her social communication has regressed. Now she is back at school she’s currently not interacting with peers.” (R234: 90)

A less prominent but nevertheless recurring theme in the free text responses was the positive effects of staying at home during the pandemic. Respondents still expressed concern that their children were falling behind educationally, but reported that their child had benefitted in terms of social skills and prolonged family contact:

“I fear my son’s learning will have regressed significantly, particularly maths. On the positive side his life skills have improved and we have enjoyed lots of time as a family.” (R179: 91)

Elsewhere, parents noticed improvements due to increased time interacting with siblings. One respondent noticed their daughter’s speech and creative play had developed due to increased one-on-one time with her sister. Another noticed improvements for the same reasons, contrasting it with her daughter feeling pressure to interact with peers at school:

“[She’s] much happier without the stress of having to conform.” (R136: 91)

Similarly, a parent/carer reported that their son was:

“flourishing and taking good learning risks because he’s not under the microscope or the ‘evaluative gaze’ at home.” (R150: 91)

Another respondent recounted how their son’s school had noticed how much progress he had made during the first lockdown:

“I put this down to us being at home all day […] so he got established in routines and play activities in a way he hadn’t before with typical family busyness.” (R200: 90)

In this instance, the parent/carer also reported that the school were posting videos of activities on Twitter and Instagram and via an online learning journal very effectively and supporting parents/carers in setting up appropriate activities at home. In one instance, a parent mentioned paying for tutorials tailored specifically for children with Down syndrome, which was viewed as an excellent resource, stating:

“[It’s] just a shame that we can’t look at funding this for all pre-school children with DS, now that location is not a barrier.” (R195: 90)

### 3.4. Impact of COVID-19 measures on access to education

Thirty-five (76%) children had not attended their educational setting during the pandemic. Of those with mainstream placements, 25% (*n* = 5) were attending school, and of those with special school places, 36% (*n* = 5) were attending school (see Table 2). Of the children who were not attending school, 17 (48.6%) parents/caregivers reported their child was frequently missing being at school and 19 (52.3%) reported finding the experience of home-schooling difficult. Seventeen (48.6%) parents reported receiving some support from school to home-school their child. One respondent expressed frustration that the school appeared to have forgotten that her daughter’s work needed to be differentiated or tailored to her needs:

“I just feel that schools not having any work to give pupils that need an element of contact with other pupils was a big let-down and caused social isolation. Group work or a group online session would have helped this.” (R151: 91)

This parent concluded that their daughter would not have done any work at all had they not been a trained Teaching Assistant themselves and therefore able to personally provide the specialist support required at home. A very similar experience was recounted by another parent who described how the work given to their son was not differentiated to his level. Another respondent wanted their child’s school to do more live online teaching so their child could see his peers rather than short videos which were not easy to engage with.

Parents reflected on the challenges of home working, home schooling and lack of support engendered by the lockdowns:

“Families who are expected to work from home with children with special needs have been effectively made to neglect their children. It has been a horrendous year for us as a family […] I am now furloughed which has made a big difference, but back at work soon with no childcare or support.” (R174: 91)

### 3.5. Impact of COVID-19 measures on access to community services

**Figure 1:**
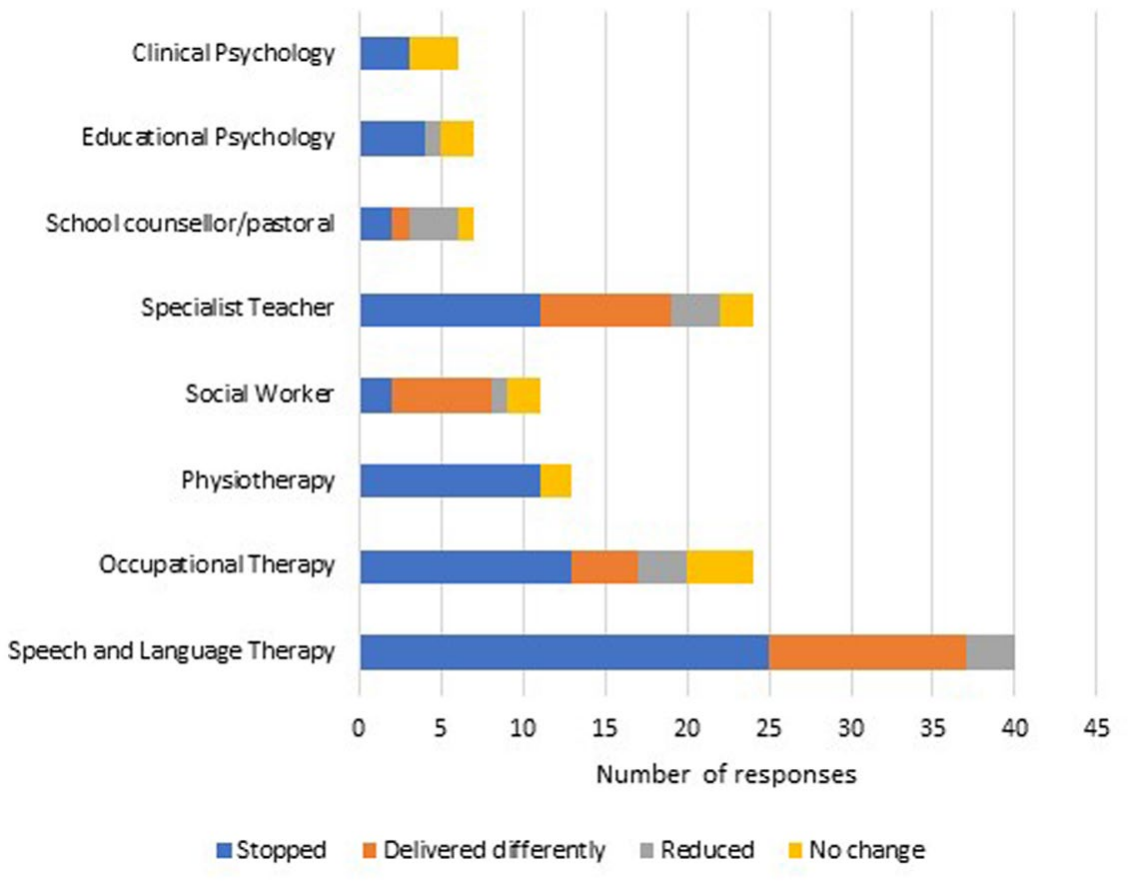
Change in delivery of specialist support during the COVID-19 pandemic.

Figure 1 shows that many support services stopped, were delivered differently or reduced during the COVID-19 pandemic. All CYP with Down syndrome receiving speech and language therapy experienced a change in delivery in the months prior to completing the questionnaire: 63% (*n* = 25) reported that their speech and language therapy provision was stopped, 30% (*n* = 12) reported it was delivered in a different way and 7% (*n* = 3) reported it was reduced. Eighty-three percent (*n* = 20) of those receiving occupational therapy experienced a change in delivery: 54% (*n* = 13) reported that it had stopped, 17% (*n* = 4) that it was delivered differently and 13% (*n* = 3) that it had reduced. Physiotherapy and clinical psychology were either stopped (*n* = 11, 85% and *n* = 3, 50% respectively) or continued without change (*n* = 2, 15% and *n* = 3, 50% respectively).

Beyond health services, 54% (*n* = 13) of those receiving support from specialist teachers continued to receive this assistance, mostly in a different way. Educational psychology support was either stopped (*n* = 4, 57%), reduced (*n* = 1, 14%) or continued unchanged (*n* = 2, 28%). Of all support services, social work and school counseling/pastoral care were most likely to continue during the COVID-19 pandemic (*n* = 9, 82% and *n* = 5, 71% respectively), although note the small numbers of families accessing these services (see Figure 1).

Twenty-seven parents/carers (58.6%) reported finding accessing healthcare/wellbeing-related appointments harder than usual since the onset of the COVID-19 pandemic with reasons given including canceled appointments, and difficulties accessing telehealth. The free text responses revealed that 14 parents/carers had appointments canceled with a range of services including audiology, optometry, dentistry in addition to hospital and clinic appointments. Two parents/carers were able to access health advice over the phone or via an online appointment, for example by speaking to a Community Specialist Health Visitor. Another respondent had organized an online meeting but had problems with their internet connection. One parent/carer was informed that they could not access speech and language therapy online because the service did not have laptops available for videocalls. Some respondents reported care being delayed, for example an annual blood test.

For some, personal circumstances made it very challenging to attend appointments. One parent/carer described how difficult it was to make an appointment because their son is a vulnerable adult; one parent/carer could not attend appointments because their child would not wear personal protective equipment (PPE). Childcare was an issue, with some parent/carers struggling to find childcare for younger siblings. Concerns over catching COVID-19 or shielding prevented further parent/carers from arranging appointments. Another parent had tried unsuccessfully to speak to a GP or pediatrician about their child’s COVID risk level. For those who were offered online appointments, respondents reported problems with their children engaging with videocalls.

Overall, 25 of the respondents (60%) reported that they rarely or never received support for their child when they needed it during the pandemic. Parents reported that support regarding their child’s learning (*n* = 32, 70%), speech (*n* = 23, 50%), language (*n* = 21, 46%), social skills (*n* = 21, 46%), emotional status (*n* = 19, 41%) and health/physical development (*n* = 14, 30%) would have been helpful. When asked for their preferences regarding support in the context of a pandemic, most parents/carers reported their preference for receiving support and information from a professional (*n* = 39, 85%) and in some cases, support from a professionally trained parent (*n* = 15, 33%) or a community of parents (*n* = 15, 33%). When asked what form they would prefer to receive help, parents stated by videocall (*n* = 24, 52%), online materials (*n* = 22, 48%), WhatsApp/text (*n* = 13, 28%), online videos (*n* = 13, 28%), written materials (*n* = 9, 20%), podcasts (*n* = 8, 17%), and online helplines (*n* = 7, 15%). Few parents selected the following as helpful support tools: a telephone helpline (*n* = 4, 9%) books/pamphlets (*n* = 2, 4%) or via the media (*n* = 1, 2%).

## 4. Discussion

To our knowledge, this is the first study to report on a wide range of impacts of COVID-19 measures on speech, language and communication abilities, social, and emotional functioning, mental health and changes in support services during the pandemic for CYP with Down syndrome specifically. Parents reported concerns about the negative impacts of COVID-19 restrictions across all domains. There were high levels of concern reported for learning, communication skills and social and emotional functioning and observations of deterioration in domains known to be areas of need associated with Down syndrome, such as expressive language and production of speech sounds/phonology and attention (Abbeduto et al., 2007; Grieco et al., 2015; Laws et al., 2015). Deterioration in wider domains related to social and emotional wellbeing and behavior, including greater reliance on adults and concerns about deterioration in social skills were also reported for some CYP with Down syndrome. This is consistent with other studies reporting data from parents/carers of children with SEND during the COVID-19 pandemic. In their sample of 241 parents of children with SEND, Asbury et al. (2021) reported parental worry about their children falling further behind in school as well as changes in behavior and low mood, in some cases severe challenging behavior. Broader impacts of COVID-19 have also been reported for adults with Down syndrome, including reduced independence and greater social withdrawal (Villani et al., 2020).

Relatively low levels of awareness of COVID-19 and worries related to COVID-19 were reported for CYP with Down syndrome. Moreover, there was a relatively small proportion of CYP with Down syndrome for whom parents/carers reported raised levels of anxiety since the onset of the pandemic (24%). This is in comparison with 73% in the remainder of the sample of CYP with SEND recruited as part of the larger study (Joffe et al., in preparation; Sideropoulos et al. 2022b) compared levels of anxiety for CYP with Down syndrome with CYP with other types of SEND, reporting lower levels of anxiety in CYP with Down syndrome, consistent with findings from this study. In line with these results, a population study (Van Gameren-Oosterom et al., 2011) has also reported lower levels of anxiety in children and young people with Down syndrome compared with other types of SEND and the general population. Increased rates of anxiety, however, have been reported in adults with Down syndrome (Vicari et al., 2013; Malegiannaki et al., 2019), and the younger age range of the sample in the present study may therefore also partly explain the lower levels of anxiety.

Anxiety was predicted by awareness of COVID-19 for both CYP with Down syndrome, and CYP with SEND (Sideropoulos et al., 2022a,b). Therefore, it is possible that the low awareness of COVID-19 reported by the parents/carers in our study could explain the lower rates of raised anxiety during the pandemic also reported. Moreover, low levels of awareness of COVID-19 may be in part explained by the difficulties parents/carers reported in explaining the pandemic and measures associated with COVID-19 to their CYP with Down syndrome. Asbury et al. (2021) report, that for children with a broad range of SEND, a lack of understanding and awareness of COVID-19 may have impacted on children with SEND differently, in some cases, resulting in lower levels of anxiety, and in others greater levels of anxiety, distress and challenging behavior due to a lack of understanding of the measures families were experiencing at that time. Parents/carers reported using a range of means to explain COVID-19 measures to their children, including social stories, telling stories and information provided by schools. Toseeb et al. (2020) similarly reported that some parents/carers of CYP with SEND felt that access to social stories to help explain the pandemic to their child with communication needs would have been helpful.

Most of the children with Down syndrome were not attending school during this period of the COVID-19 pandemic. Parents/carers reported concerns about meeting their child’s needs and finding home-schooling difficult. Similarly, Sideropoulos et al. (2022a) reported higher levels of worry about school closures and change in routine for CYP with SEND compared with their typically developing siblings, and difficulties with home-schooling for CYP with SEND have been reported across a number of studies (O’Hagan and Kingdom, 2020; Toseeb et al., 2020; Asbury et al., 2021). Asbury et al. (2021) found that parents of children with SEND reported feelings of distress and being overwhelmed by meeting their child’s needs during school closures. Toseeb et al. (2020) suggest that this level of concern is likely to be much higher for parents of CYP with SEND due to their complex educational needs (although Toseeb et al. did not directly compare children with SEND with children without SEND). These findings have clear implications for additional education support needed by CYP and their parents/carers in the context of school closures.

High numbers of parents/carers reported a reduction, cessation or change in service delivery of specialist support services, including therapy services, psychology and specialist education services. This is consistent with reports of inadequate levels of support for children with SEND during COVID-19 (O’Hagan and Kingdom, 2020; Toseeb et al., 2020). Fifty percent of a sample of over 4000 parents/carers of CYP with SEND surveyed in May 2020 reported that external therapies had stopped (Disabled Children’s Partnership, 2020). Community children and young people’s services were particularly impacted by the COVID-19 pandemic at the time of this study, due to redeployment of staff to front-line services and changes in the mode of service delivery (Chadd et al., 2021; NHS Confederation and NHS Providers, 2022). This reduction in access to support services for CYP with Down syndrome, as for all children with SEND, arguably at a time of increased need, will have put them at increased risk of poorer outcomes, for example academic attainment, and difficulties with friendships, social functions and mental health (Royal College of Speech and Language Therapists, 2021). A deterioration in reading and writing skills, reported by 22% of parents/carers, suggests that education and speech and language therapy professionals may also need to pay particular attention to providing additional support to enable CYP with Down syndrome to reach their full potential in terms of literacy skills post-pandemic. Enhancing phonological awareness, decoding and providing positive home literacy environments have all been shown to be important and beneficial for promoting literacy in CYP with Down syndrome (Loveall and Barton-Hulsey, 2021).

Parents also reported that additional support for learning, speech and language, social–emotional and physical health would have been helpful during the pandemic, reflecting the complex pattern of needs and number of support services required for CYP with Down syndrome. This is similar to the finding of Toseeb et al. (2020), who found that parents/carers of CYP with SEND reported the need for ongoing support tailored to their child’s needs as well as professional advice and support from teaching staff and therapies (Toseeb et al., 2020). It should be noted that in our study parents reported that social work and school counseling/pastoral care was much less likely to be stopped than other education and health services. It is interesting to consider why this was the case and how it was possible for some services to continue throughout the pandemic relatively unchanged. Learning and experiences from some services may be helpful in considering how other support services respond to a pandemic situation in the future.

It should also be noted that positive impacts of the COVID-19 measures were reported by some parents/carers of CYP with Down syndrome. These included increased time spent with siblings and family, improved play, communication and life skills. This is also consistent with findings from Asbury et al. (2021) who reported that, for some children with SEND, positive emotions were reported for children who found being home a respite from school, and O’Connell et al. (2020) who found that some parents reported improved quality time, increased family time and benefits to learning.

The present study measured changes in speech, language and communication abilities, behavior, social and emotional functioning, and mental health and access to education and healthcare services using a bespoke questionnaire and caregiver report rather than pre-existing validated measures. It is important that future work considers using validated caregiver measures to further explore associations between speech, language and communication abilities, and social emotional and behavioral outcomes in this population. This study focused on caregiver report of child outcomes rather than caregiver burden, stress and anxiety. An understanding of the impact of pandemic measures on caregivers, using measures such as the Caregiving Difficulty Scale (McCallion et al., 2005) would provide further insights. In addition the sample included parents of CYP with Down Syndrome across a wide age range. Due the relatively small sample size it was not possible to look at differences between age groups, but this would be important to explore in further studies with this population.

## 5. Conclusion

These findings highlight the wide-ranging impacts of COVID-19 on CYP with DS and their families, both in terms of impacts on learning, speech, language, communication and literacy as well as social, emotional and behavioral domains, and in terms of a lack of support from schools and community services. These findings have implications for the support that is now needed for CYP with Down syndrome and their families, who have experienced reduced provision throughout the pandemic, and important lessons for periods of social restrictions in the future. The high level of parental/carer concern about meeting their child’s needs through home-schooling suggests the need for additional and individualized support for families of CYP with Down syndrome both during periods of school closures and also post-pandemic. Clear preferences to receive support from professionals, trained parents or a community of parents provide insights into new ways in which families can be supported, by widening the ‘expert team’ to include parents/carers and parent communities. Parents also seemed happy to receive support and training through a range of mediums, including online and virtual delivery and dissemination, making it possible for alternative solutions adopted as a result of the pandemic, and acceptable to families, to be incorporated into future mainstream provision of support.

## Data availability statement

The raw data supporting the conclusions of this article will be made available by the authors, without undue reservation.

## Ethics statement

 Ethical approval was granted by the Faculty of Science and Health Ethics Sub-Committee at the University of Essex before the start of the study. The patients/participants provided their written informed consent to participate in this study.

## Author contributions

EP, PH, and VJ contributed to the conception and design of the study. EP and RD organized the database. EP conducted the quantitative analysis. RD conducted and wrote up the content analysis. EP wrote the first draft of the manuscript. All authors contributed to manuscript revision, read, and approved the submitted version.

## Conflict of interest

The authors declare that the research was conducted in the absence of any commercial or financial relationships that could be construed as a potential conflict of interest.

## Publisher’s note

All claims expressed in this article are solely those of the authors and do not necessarily represent those of their affiliated organizations, or those of the publisher, the editors and the reviewers. Any product that may be evaluated in this article, or claim that may be made by its manufacturer, is not guaranteed or endorsed by the publisher.
