# Supplementary material for: The impact of COVID-19 (Coronavirus) on children and young people with Down syndrome in the United Kingdom
Source: Front Psychol. 2023 Jun 2;14:1175636. doi: 10.3389/fpsyg.2023.1175636 (PMC10272386; doi:10.3389/fpsyg.2023.1175636)
Supplement: Supplementary file 1 [file Table_1.DOCX]

Survey on the impact of COVID19 (Coronavirus) on the social and emotional functioning of children and young people with Speech, Language and Communication Needs

*By ‘COVID-19 outbreak’, we mean the COVID-19 (coronavirus) illness and the government advice and restrictions that have been put in place to help prevent the spread of the illness (e.g. ‘social distancing’ measures and school and shop closures).*

*In this survey, we ask you to tell us about your child with speech, language and communication needs. If you have more than one child with difficulties in speech, language or communication, please would you select* ***one child*** *to base this survey on.*

**About you and your child**

1. **Your relationship to your child:**

| Mother |  |
| --- | --- |
| Father |  |
| Grandparent |  |
| Foster parent/carer |  |
| Other/Guardian |  |

1. ***Choose one option that best describes your ethnic background.***

| Asian / Asian British – Indian Pakistani, Bangladeshi, other | Chinese/Chinese British |
| --- | --- |
| Black / Black British – Caribbean, African, other | Middle Eastern/Middle Eastern British – Arab, Turkish, other |
| Mixed race – White and Black/Black British | Pakistani |
| Mixed race - other | Other |
| White – British/Irish/Other | Prefer not to say |

1. **Is your child’s ethnic group different from yours?**

| **Yes** |  |
| --- | --- |
| **No** |  |

**3a If yes, what is your child’s ethnic region?**

| Asian / Asian British – Indian Pakistani, Bangladeshi, other | Chinese/Chinese British |
| --- | --- |
| Black / Black British – Caribbean, African, other | Middle Eastern/Middle Eastern British – Arab, Turkish, other |
| Mixed race – White and Black/Black British | Pakistani |
| Mixed race - other | Other |
| White – British/Irish/Other | Prefer not to say |

1. **Where do you live?**

| Scotland |  |
| --- | --- |
| Northern Ireland |  |
| Wales |  |
| North East England |  |
| North West England |  |
| Yorkshire and the Humber |  |
| West Midlands |  |
| East Midlands |  |
| South West England |  |
| South East England |  |
| East of England |  |
| Greater London |  |
| USA/North America |  |
| South America |  |
| Asia |  |
| Africa |  |
| Europe (other than the UK) |  |
| Australia/NZ |  |

1. **Who usually lives in your household, in addition to your child?**

|  |  |
| --- | --- |
| **Mother** |  |
| **Father** |  |
| **Grandmother** |  |
| **Grandfather** |  |
| **Foster parent/carer** |  |
| **Other adults (any person age 19 or above)** |  |
| **Siblings** |  |

1. **What is your child’s gender?**

| Male |  |
| --- | --- |
| Female |  |
| Other/prefer not to say |  |

1. **How old is your child?**

| 1 |  |
| --- | --- |
| 2 |  |
| 3 |  |
| 4 |  |
| 5 |  |
| 6 |  |
| 7 |  |
| 8 |  |
| 9 |  |
| 10 |  |
| 11 |  |
| 12 |  |
| 13 |  |
| 14 |  |
| 15 |  |
| 16 |  |
| 17 |  |
| 18 |  |
| 19 |  |
| 20 |  |
| 21 |  |
| 22 |  |
| 23 |  |
| 24 |  |
| 25 |  |

**8. What type of School does your child attend?**

| Childminder |  |
| --- | --- |
| Nursery |  |
| State Mainstream Preschool |  |
| State Mainstream Primary School |  |
| State Mainstream Secondary School |  |
| Private Mainstream Preschool |  |
| Private Mainstream Primary School |  |
| Private Mainstream Secondary School |  |
| Special Preschool |  |
| Special Primary School |  |
| Special Secondary School |  |
| Specialist Language Resource in a Mainstream Preschool |  |
| Specialist Language Resource in a Mainstream Primary School |  |
| Specialist Language Resource in a Mainstream Secondary School |  |
| Home Educated |  |
| Further Education College |  |
| University |  |

**About your child’s speech, language and communication needs**

1. **Does your child have a Special Educational Needs and Disability Diagnosis?**

| Yes |  |
| --- | --- |
| No |  |

**9a If yes, what is the diagnosis?**

(please tick all that apply)

| Autistic Spectrum Disorder |  |
| --- | --- |
| Cerebral Palsy/physical disability |  |
| Downs Syndrome |  |
| William’s Syndrome |  |
| Developmental Language Disorder |  |
| Language Disorder |  |
| Attention Deficit Hyperactivity Disorder |  |
| Dyspraxia |  |
| Dyslexia |  |
| Hearing Impaired |  |
| Moderate Learning Difficulty |  |
| Sever Learning Difficulty |  |
| Profound and Multiple Learning Difficulty |  |
| Cleft Lip and/or Palate |  |
| Visual Impairment |  |
| Social Communication Disorder |  |
| Selective Mutism |  |
| Other (please specify?) |  |

1. **Does your child have an Educational and Health Care Plan or some other Statement of Special Education Need?**

| Yes |  |
| --- | --- |
| No |  |

1. **What type of speech, language and communication difficulty does your child have?** *(please tick all that apply)*

| Difficulties with pronunciation/production of speech sounds |  |
| --- | --- |
| Stammering/Stuttering |  |
| Difficulties with talking – using words and sentences |  |
| Voice |  |
| Difficulties with understanding what other people say |  |
| Physical difficulties impairing their ability to walk and/or move and/or talk |  |
| Cleft lip and/or palate |  |
| Social Difficulties |  |
| Emotional Difficulties |  |
| Behaviour Problems |  |
| Difficulties interacting with other people |  |
| Reading and writing |  |

1. **Does your child receive any specialist support?**

| Yes |  |
| --- | --- |
| No |  |

**12a. If yes, what type of support?** (tick all that apply)

| Speech and Language Therapist |  |
| --- | --- |
| Occupational Therapist |  |
| Physiotherapist |  |
| Clinical Psychologist |  |
| Educational Psychologist |  |
| Social Worker |  |
| School Counsellor/Pastoral Co-ordinator |  |
| Specialist Teacher |  |

1. **Please select 4 to show you are reading this.**

| 1 |  |
| --- | --- |
| 2 |  |
| 3 |  |
| 4 |  |
| 5 |  |
| 6 |  |
| 7 |  |
| 8 |  |

**About your child and COVID19**

1. **Has your child been attending School during the COVID19 period?**

| Yes | (go to XX) |
| --- | --- |
| No | (go to XX) |

**14a If no, how often is your child missing being at School?**

| Always |  |
| --- | --- |
| Often |  |
| Sometimes |  |
| Rarely |  |
| Never |  |

**14b How have you found the experience of home schooling your child?**

| Very easy |  |
| --- | --- |
| easy |  |
| average |  |
| difficult |  |
| Very difficult |  |

**14c How much support have you received from School to home school your child**

| Lots of Support |  |
| --- | --- |
| Some Support |  |
| A little Support |  |
| No support |  |

1. **Please indicate any changes to the usual support your child receives during this COVID19period? (Tick all that apply)**

| Support | Stopped | Delivered in a different way | Reduced | No Change | N/A |
| --- | --- | --- | --- | --- | --- |
| Speech and Language Therapist |  |  |  |  |  |
| Occupational Therapist |  |  |  |  |  |
| Physiotherapist |  |  |  |  |  |
| Clinical Psychologist |  |  |  |  |  |
| Educational Psychologist |  |  |  |  |  |
| Social Worker |  |  |  |  |  |
| School Counsellor/Pastoral Co-ordinator |  |  |  |  |  |
| Specialist Teacher |  |  |  |  |  |

1. **Are you** **concerned that your child’s communication, learning and development has deteriorated during this period?**

| **Yes** |  |
| --- | --- |
| **No** |  |

**16a. If yes, what areas do you think there has been deterioration in? (tick all that apply)**

| Speech |  |
| --- | --- |
| Language |  |
| Communication |  |
| Social Skills |  |
| Attention |  |
| Memory |  |
| Behaviour |  |
| Skills for daily living? |  |
| Play |  |
| Physical Health |  |
| Emotional Wellbeing |  |

1. **How often does your child ask you questions about COVID19?**

| Always |  |
| --- | --- |
| Often |  |
| Sometimes |  |
| Rarely |  |
| Never |  |

1. **How easy is it for you to explain the current COVID19 situation to your child?**

| Very Easy |  |
| --- | --- |
| Easy |  |
| Neutral |  |
| Difficult |  |
| Very Difficult |  |

1. **My child understands what is happening during this period**

| Completely |  |
| --- | --- |
| A lot |  |
| A little |  |
| Not at all |  |

1. **What resources have you used to help your child understand the COVID19 situation? (tick all that apply)**

| Information from your child’s teacher/school |  |
| --- | --- |
| Website |  |
| Youtube clips |  |
| Children’s TV programmes |  |
| News items |  |
| Talking with friends |  |
| By telling them a story |  |
| Social Story |  |
| Other |  |

1. **Have you noticed any changes in your child since the COVID19 situation?**

| Yes |  |
| --- | --- |
| No |  |

**21a If yes, can you indicate which behaviours your child has been showing during this period.** Please only identify those behaviours which have been identified during this period, or which have increased during this period. (please tick all that apply).

| Anxiety |  |
| --- | --- |
| Aggression |  |
| Anger |  |
| Passivity |  |
| Reduced Motivation |  |
| Increased Sleeping |  |
| Reduced Sleep |  |
| Experiencing Nightmares |  |
| Withdrawn |  |
| Loss of appetite |  |
| Increased Appetite |  |
| Tearful |  |
| Moody |  |
| Quiet/Withdrawn |  |
| Disruptive |  |
| Irritability |  |
| Restlessness |  |
| Isolated/loneliness |  |
| Reduced enthusiasm |  |
| Greater dependence on adults |  |
| Increased neediness |  |
| Increased reliance on the television |  |
| Increased reliance on computer games |  |
| Other |  |

1. **My child has shown increased anxiety during the COVID19 period**

| Always |  |
| --- | --- |
| Often |  |
| Sometimes |  |
| Rarely |  |
| Never |  |

1. **Please select ‘Always’ if you are still reading the survey.**

| Always |  |
| --- | --- |
| Often |  |
| Sometimes |  |
| Rarely |  |
| Never |  |

1. **During the COVID – 19 outbreak, how often have you worried about the following in relation to your child?**

|  | Frequently | Sometimes | Rarely | Never | Unsure / does not apply |
| --- | --- | --- | --- | --- | --- |
| My child’s health |  |  |  |  |  |
| Meeting my child’s learning or development needs |  |  |  |  |  |
| My child’s speech, language or communication development |  |  |  |  |  |
| Feeding my child a healthy diet |  |  |  |  |  |
| Enjoying relaxed time with my child |  |  |  |  |  |
| My child’s emotional wellbeing |  |  |  |  |  |
| The amount of exercise my child is getting |  |  |  |  |  |
| My child’s social interactions |  |  |  |  |  |
| My child’s behaviour |  |  |  |  |  |

1. **My child is missing interacting with his friends**

| Always |  |
| --- | --- |
| Often |  |
| Sometimes |  |
| Rarely |  |
| Never |  |

1. **My child is missing his grandparents**

| Always |  |
| --- | --- |
| Often |  |
| Sometimes |  |
| Rarely |  |
| Never |  |
| N/A |  |

1. **My child is able to interact with friends and family over the computer/tablet/IPad/phone**

| Always |  |
| --- | --- |
| Often |  |
| Sometimes |  |
| Rarely |  |
| Never |  |

1. **During this period, my child has expressed the following fears. (tick all that apply**

| Fear of dying |  |
| --- | --- |
| Getting ill |  |
| Losing parents |  |
| Losing grandparents |  |
| Other |  |

**29. During the COVID-19 outbreak, have you found it more difficult than usual to attend or access health/wellbeing related appointments for your child)?**

e.g. doctor’s appointment, speech and language therapy. This may be in person or online.

| Yes | No (Go to XX) |
| --- | --- |
| Does not apply (I have not had any appointments) (Go to XX) |  |

**29b If yes, who was this appointment(s) with?**

**29c Please briefly state why it has been more difficult (e.g. can’t get to appointment, trouble with internet access)**

**Help and support during COVID-19**

1. **During COVID19, I received support for my child when I needed it to help them cope with the current situation**

| Always |  |
| --- | --- |
| Frequently |  |
| Sometimes |  |
| Rarely |  |
| Never |  |

**30a If yes, can you describe the support you received?**

1. **What areas of support would have been helpful for you during this period in supporting your child?**

|  | Yes | No |
| --- | --- | --- |
| My child’s behaviour |  |  |
| My child’s diet |  |  |
| My child’s social development |  |  |
| My child’s emotional wellbeing |  |  |
| My child’s speech development |  |  |
| My child’s language development |  |  |
| My child’s learning |  |  |
| My child’s exercise |  |  |
| My child’s response to the Covid-19 pandemic (e.g. their understanding of the pandemic or their worries about it) |  |  |
| My child’s health/physical development |  |  |

If you answered no, please go to question

If you answered yes, please go to question

**30a. If yes, from whom would you prefer to receive help, support or information?**

| From a professional / professional service | From another parent who is professionally trained in supporting others |
| --- | --- |
| From a community of parents in similar circumstances |  |

**30b. In what form would you prefer to receive help, support or information?**

| Online written materials | On paper written materials |
| --- | --- |
| Online videos | Podcasts |
| Telephone helpline | Online helpline |
| WhatsApp (text) | Telephone call by appointment |
| Videocall (e.g. Skype, Zoom) | Other. Please state |

1. **Is there anything else you would like to tell us about the impact that COVID19 is having on your child’s social and emotional development?**

**Thank you for your contribution**
